# Lanthanum Recovery from Aqueous Solutions by Adsorption onto Silica Xerogel with Iron Oxide and Zinc Oxide

**DOI:** 10.3390/gels11050314

**Published:** 2025-04-23

**Authors:** Ionuţ Bălescu, Mihaela Ciopec, Adina Negrea, Nicoleta Sorina Nemeş, Cătălin Ianăşi, Orsina Verdes, Mariana Suba, Paula Svera, Bogdan Pascu, Petru Negrea, Alina Ramona Buzatu

**Affiliations:** 1 Faculty of Chemical Engineering, Biotechnologies and Environmental Protection, Politehnica University Timişoara, Victoriei Square, No. 2, 300006 Timisoara, Romania; ionut.balescu@student.upt.ro (I.B.); adina.negrea@upt.ro (A.N.); petru.negrea@upt.ro (P.N.); 2 Research Institute for Renewable Energies—ICER, Politehnica University Timişoara, Gavril Musicescu Street, No. 138, 300774 Timisoara, Romania; nicoleta.nemes@upt.ro (N.S.N.); ioan.pascu@upt.ro (B.P.); 3 Coriolan Drăgulescu’ Institute of Chemistry, Mihai Viteazul Bvd., No. 24, 300223 Timisoara, Romania; orsinaverdes@acad-icht.tm.edu.ro (O.V.); mariana_suba@acad-icht.tm.edu.ro (M.S.); 4 National Institute for Research and Development in Electrochemistry and Condensed Matter-Timisoara—INCEMC, Dr. A. Păunescu Podeanu Street, No. 144, 300569 Timisoara, Romania; paulasvera@gmail.com; 5 National Research and Development Institute for Welding and Material Testing—ISIM, Timişoara, Mihai Viteazul, Bvd., No. 30, 300222 Timisoara, Romania; 6 Department of Biochemistry and Pharmacology, “Victor Babeș” University of Medicine and Pharmacy, Eftimie Murgu Square No. 2, 300041 Timisoara, Romania; buzatu.ramona@umft.ro

**Keywords:** lanthanum, adsorption, xerogel, composite, silica, iron oxide, zinc oxide

## Abstract

From the lanthanide group, part of the rare earth elements (REEs), lanthanum is one of the most important elements given its application potential. Although it does not have severe toxicity to the environment, its increased usage in advanced technologies and medical fields and scarce natural reserves point to the necessity also of recovering lanthanum from diluted solutions. Among the multiple methods for separation and purification, adsorption has been recognized as one of the most promising because of its simplicity, high efficiency, and large-scale availability. In this study, a xerogel based on silicon and iron oxides doped with zinc oxide and polymer (SiO_2_@Fe_2_O_3_@ZnO) (SFZ), obtained by the sol–gel method, was considered as an adsorbent material. Micrography indicates the existence of particles with irregular geometric shapes and sizes between 16 μm and 45 μm. Atomic force microscopy (AFM) reveals the presence of dimples on the top of the material. The specific surface area of the material, calculated by the Brunauer–Emmet–Teller (BET) method, indicates a value of 53 m^2^/g, with C constant at a value of 48. In addition, the Point of Zero Charge (pH_pZc_) of the material was determined to be 6.7. To establish the specific parameters of the La(III) adsorption process, static studies were performed. Based on experimental data, kinetic, thermodynamic, and equilibrium studies, the mechanism of the adsorption process was established. The maximum adsorption capacity was 6.7 mg/g, at a solid/liquid ratio = 0.1 g:25 mL, 4 < pH < 6, 298 K, after a contact time of 90 min. From a thermodynamic point of view, the adsorption process is spontaneous, endothermic, and occurs at the adsorbent–adsorbate interface. The Sips model is the most suitable for describing the observed adsorption process, indicating a complex interaction between La(III) ions and the adsorbent material. The material can be reused as an adsorbent material, having a regeneration capacity of more than 90% after the first cycle of regeneration. The material was reused 3 times with considerable efficiency.

## 1. Introduction

In nature, lanthanum is found together with other rare earth elements (REEs), called lanthanides [1,2,3,4], being quite abundant in the Earth’s crust [5]. In 2008, lanthanum represented approximately 30% of the total amount of REEs used. Around the year 2010, Japan and the United States ranked second and third, respectively, in terms of global consumption of rare earth materials [6]. A forecast of the total latent demand for lanthanum oxide globally (excluding China) for 2025 is 14,382 tons [7]. Currently, the largest consumer of REEs is China, with their primary usage focused on the manufacturing of electronic products.

Lanthanum compounds are used in many industrial segments, e.g., as catalysts in petroleum cracking processes [6,7], as well as in the manufacture of catalytic converters for the automotive industry [8,9]. In the glass industry, lanthanum and other REE compounds are widely used, either as additives for the absorption of ultraviolet light or as modifiers of the refractive index for coloring/discoloring of glass [10,11], and can increase the material’s hardness, allowing the use of glass powder as a polishing powder. In addition, lanthanum is added in trace amounts to steel, iron, aluminum, and other metal alloys to enhance specific physical characteristics. Regarding the usage of lanthanum compounds in optics, it can be used in the production of phosphorescent screens with cathode ray tubes, fluorescent lamps, and other applications that require the production of colored light [12,13]. There is also a growing demand for lanthanum in special industries. Thus, in the electronics industry, lanthanum is added to the composition of ceramic glazes or enamels for color control and is also used in larger quantities to manufacture the negative electrodes of some types of rechargeable batteries, such as nickel–metal–hydrides [14]. Some lanthanum-based compounds are used in water treatment, e.g., lanthanum hydroxide can be used for water defluorination [15] or to remove arsenic compounds from deep waters [16].

Regarding the presence of lanthanum and other REEs in the environment, this is due to their persistence in the soil, as well as to their migration through groundwater [17]. In the atmosphere, lanthanum concentrations are usually very low (0.05–30 μg/m^3^) even in urban areas with high levels of pollution [18,19].

As with other metals, the availability of lanthanum is strongly influenced by pH and the presence of other cations in the environment [20,21]. It can accumulate in organisms, interfere with cellular functions, and be adsorbed by various other particles [22]. High concentrations of lanthanum have been found in the kidneys, livers, and lungs of workers in smelters, printing presses, and refineries [23,24,25], as well as in the case of people responsible for film projection and cinema operation [26,27].

One surface process specific to La(III) retention is adsorption. Silica xerogels are currently the most widely studied group of aerogels and are of great interest in the field of wastewater treatment with the purpose of recovery or elimination of metal ions [28,29,30,31]. Xerogels have a large surface area, mainly due to the presence of mesopores. They exhibit a higher adsorption capacity compared to other commonly used adsorbents, with a high mechanical strength. They have been proven to be good adsorbent materials when their surface is modified with iron nanoparticles or other metals that form oxides or hydroxides [32]. There are multiple methods for synthesizing xerogels, and the most suitable for the synthesis of metal oxide nanocomposites are sol–gel methods. Sol–gel is widely used in practice due to its advantages in comparison to traditional methods [33]: (i) the possibility of obtaining very pure and homogeneous materials, as well as compositions that cannot be prepared by conventional methods; (ii) the possibility of creating a uniform, predetermined porosity, as well as controlling the shapes and sizes of the particles of the finished product by controlling the synthesis conditions; (iii) the simplicity of the purification of metal alkoxides (in the case of the synthesis of xerogels by hydrolysis and polycondensation of metal alkoxides); (iv) the high degree of homogeneity (at the molecular scale) in a multicomponent system; (v) a considerable decrease in the energy consumption necessary for the sintering of colloidal particles, due to the high surface energy that favors sintering at lower temperatures; (vi) the possibility of producing nanocrystalline systems, whose synthesis by traditional methods leads to phase separation or crystallization; (vii) the possibility of obtaining organic–inorganic hybrid materials.

However, there are also disadvantages to the sol–gel method: (i) mechanical fragility (xerogels are often brittle materials, with low mechanical strength, which can limit their use in applications involving mechanical stress); (ii) production costs (the process of preparing xerogels can be complex and expensive, especially for materials that require controlled porosity); (iii) moisture sensitivity (some xerogels can be sensitive to moisture, which can affect their properties); (iv) volume shrinkage (during the drying process, xerogels can undergo significant volume shrinkage, which can lead cracking of the material).

However, the synthesis of materials based on silicon and iron oxides doped with zinc oxide remains a viable alternative when aiming to obtain a porous adsorbent material to be used for the recovery of metal ions from aqueous solutions.

In this context, the novelty and the objectives of this study were as follows: (i) synthesis of SiO_2_, Fe_2_O_3_-doped ZnO xerogel for efficient recovery of La(III) from aqueous media, by the sol–gel synthesis method; (ii) physicochemical characterization of SFZ; (iii) evaluation of the mechanism of La(III) recovery by adsorption, establishing the process mechanism through kinetic, thermodynamic and equilibrium studies starting from the specific parameters of the adsorption process; (iv) evaluating the long-term performance of the stability of the SFZ adsorbent material by performing repeated adsorption–desorption cycles.

## 2. Results and Discussion

### 2.1. Characterization of SFZ

#### 2.1.1. Thermogravimetry and Differential Thermogravimetry (TG–DTG), Raman and Fourier Transform Infrared Spectroscopy (FT–IR) Methods of Investigation

**Figure 1:**
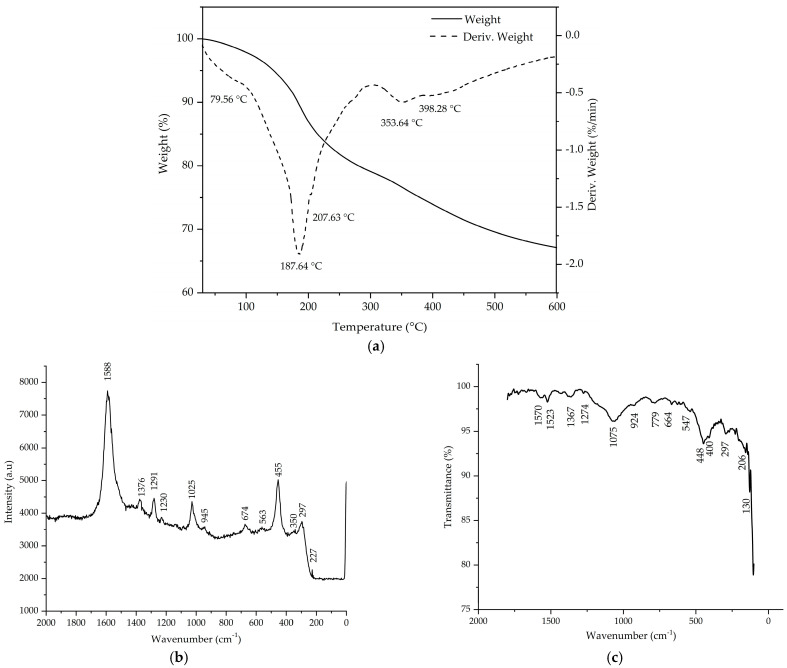
Thermogravimetry and Differential Thermogravimetry (TG–DTG) (**a**). Raman spectroscopy (**b**) and Fourier transform Infrared spectroscopy (FT–IR) (**c**) for SFZ material.

TG–DTG analysis was used to investigate the thermal stability of the SFZ sample (Figure 1).

TG–DTG analysis was used to investigate the thermal stability of the SFZ sample. According to the TG curve in Figure 1a, the total weight loss recorded is 35.7% of the weight taken. Removal of physiosorbed water and other solvents from the sample matrix is responsible for the mass loss in the range of 25–110 °C (endothermic peak observed at ~79 °C) [34,35]. The elimination of chemosorbed water and decomposition of acetylacetonate group is associated with a significant weight loss of 18% in the range of 110–200 °C (endothermic peak observed at ~187 °C) [36,37]. For the temperature range 200–250 °C, a mass loss occurs with an endothermic effect observed at 207 °C, which we presume to be specific for the partial decomposition of polyethylene glycol. Finally, the two endothermic peaks (~353 °C and 398 °C) present in the temperature range from 250 to 600 °C are attributed to the decomposition of acetylacetonate groups from the metalorganic complex and polycondensation of the silanol groups [38,39,40].

FT–IR and Raman spectroscopy are presented in Figure 1b and Figure 1c, respectively. After an initial evaluation, most of the bands that appear in the FT–IR spectrum also appear in the Raman spectrum. Since the same type of vibrational changes are associated with both IR and Raman, the resulting spectra of both methods for a given species may resemble each other. However, there are differences when it comes to which groups are IR active and which are Raman active, since the change in dipole or charge distribution is associated with IR, followed by the interaction of the same frequency between the radiation and molecule, resulting in an excited vibrational state, while Raman scattering involves a momentary distortion of the electron distribution around a bond in a molecule, followed by re-emission of the radiation as the bond returns to it ground state. The distortion itself creates a temporary dipole due to polarized molecules, which disappears upon relaxation. Since each band in a Raman spectrum has resulted from the interaction between incident light and certain atomic vibrations, any changes that appear regarding the size, valences, masses, bond forces, symmetry, or the arrangement in the crystal structure of atomic vibrations are visible [41]. From the evaluated data of the FT–IR spectrum, it is observed that the specific bands of acetylacetonate ν(C=O) and ν(C=C) are present at 1573 cm^−1^ and 1523 cm^−1^ [42]. Given the aspect of the Raman peak located at 1588 cm^−1^, it is possible that the ν(C=O) and ν(C=C) overlap, since both characteristic peaks are in the same region, as suggested in previous work [43]. The weaker bands observed at 1376 cm^−1^ and 1291 cm^−1^ in the Raman, respectively 1367 cm^−1^ and 1274 cm^−1^ in the FT–IR spectrum, are specific to the ν(C-O) and δ(CH_3_) bonds [44]. The bands around 1025 cm^−1^ in the Raman spectrum and 1075 cm^−1^ in the FT–IR spectrum are attributed to the siloxane groups ν(as) Si-O-Si [45]. The bands at 945 cm^−1^ observed in Raman and 924 cm^−1^ observed in FT–IR are specific for silanol ν Si-OH bonds [46,47]. In the case of the band at 779 cm^−1^ observed only in the FT–IR spectrum, it can be attributed to M-O bonds [47,48]. The existence of the 455 cm^−1^ Raman band and, respectively, the 448 cm^−1^ FT–IR band can be attributed to both the symmetric vibration of ν(s) O-Fe-O and the bending vibration δ O-Si-O groups [47,49]. Furthermore, the appearance of the band at 409 cm^−1^ is attributed to metal oxygen bonds (ν Si-O, ν Zn-O, ν Fe-O) [48,50,51]. The presence of bands at 225, 247, 299, 564 and 674 cm^−1^ indicate the formation of α Fe_2_O_3_ [43,51,52]

#### 2.1.2. Scanning Electron Microscopy Analysis (SEM) Coupled with Energy-Dispersive X-Ray Spectroscopy (EDX)

**Figure 2:**
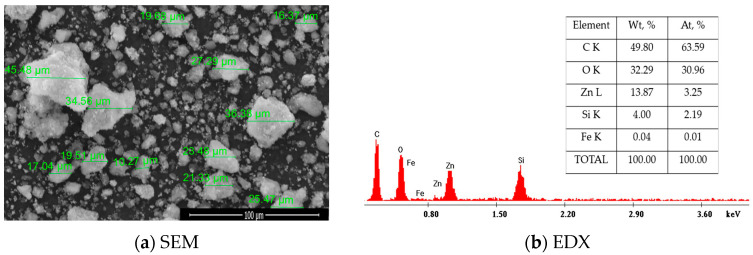
Scanning electron microscopy coupled with energy dispersive X-ray spectroscopy, SEM–EDX for SFZ material.

The morphology of SFZ is presented in an SEM image alongside the elemental composition of the synthesized nanoparticles, EDX (Figure 2a,b).

From Figure 2a, SEM micrography indicates the existence of particles with irregular geometric shapes and sizes between 16 µm and 45 µm. The majority of the present particles are separately distributed throughout the area. This is also visible with the most separately distributed particles, with a few interconnected particles forming clusters.

The SEM–EDX spectrum (Figure 2b) confirms the presence of Si, Fe, Zn and O specific peaks, certifying the formation of SiO_2_, Fe_2_O_3_ and ZnO oxides. The specific peak of C atom can be attributed both to traces of unreacted acetonate and to the carbon surface on which the sample is placed for analysis. This statement is also confirmed by the Raman and FT–IR results shown in Figure 1b,c, certifying the presence of the Si, Fe and Zn oxides alongside the traces of the carbon and oxygen bonds.

#### 2.1.3. Atomic Force Microscopy (AFM)

**Figure 3:**
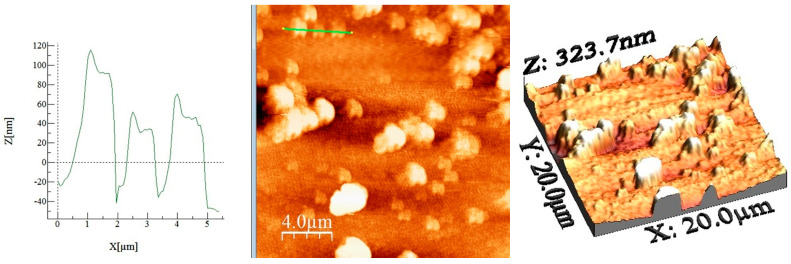
Atomic force microscopy profile images on the selected areas for SFZ material (left image). AFM 2D (middle image) and AFM 3D (right image) for SFZ material.

The AFM technique, revealing a material’s morphology and surface roughness, is a useful tool for the characterization of the material in combination with SEM and BET, since surface morphology plays a significant effect in adsorption. AFM (2D and 3D) images, surface profiles for selected areas, and roughness data were obtained and presented in Figure 3 and Table 1.

Calculated values from the AFM image (Average roughness (Sa), Mean Square Root Roughness (Sq), Maximum peak height (Sp), Maximum valley depth (Sv), Maximum peak-to valley height (Sy), Surface kurtosis (Sku), Surface skewness (Ssk)) are shown in Table 1.

The 2D AFM image in Figure 3 (middle image) reveals formations of similar morphology patterns with a specific dimple, as shown in the additional profile (left image). The multiple sizes of the material’s particles are also highlighted in the case of SEM analysis (Figure 2a), as well as their distribution. In addition to the material’s separately distributed particles, few clusters are present. However, since the image scale is different in SEM analysis, displaying larger areas, AFM provides more details regarding the smaller-sized particles that are present in a 20 × 20 μm area. Concerning the roughness value, both Sa and Sq are used to describe this, but their value differs due to the distinct calculation formulae. Their importance is also depicted in several studies, where both are used to describe height variations, but in the case of Sa there is an additional property describing average/absolute deviation of the surface irregularity from the mean line over one sampling length [53,54,55]. Additional information regarding the surface is represented by the Sku and Ssk values. More precisely, when the height distribution is symmetrical, the skewness is zero. Other situations indicate asymmetrical height distribution, implying a positive Ssk value as a result of peak prevalence and, respectively, a negative Ssk value in the case of valley prevalence. Kurtosis is used to describe the density sharpness of a profile, exhibiting high peaks and, respectively, deep valleys, giving further insight regarding the surface aspect. As well as in the case of Ssk, the value of the Sku can indicate whether the surface is “flat” (Sku < 3) or “sharp” (Sku > 3). In addition, Sp and Sv are other parameters indicating the height of the highest peak and, respectively, the largest pit within the area. Sy is defined as the sum of the largest peak’s height value (Sp) and the largest pit’s depth value (Sv) within the defined area [56]. The results obtained highlight the prevalence of peaks indicated by the Ssk value, in addition to the higher Sp value in comparison to the Sv. Additionally, Sku is >3, demonstrating a “sharper” surface due to the presence of sharp peaks. The results obtained are in concordance with the profile images of the selected areas, indicating the presence of abrupt sharp edges. As mentioned earlier, the dimples observed on top of the samples are possibly acting as traps in case of an adsorption process. The nitrogen adsorption–desorption isotherm data evaluated demonstrates the surface adsorption ability of the material, which can be correlated with the AFM asperity results. In addition, the general tendency of the surface towards asperity, expressed by the Sku and Skk values, is in accordance with the SAXS values.

#### 2.1.4. Specific Surface Area Evaluated by BET (Brunauer–Emmett–Teller) Method

**Figure 4:**
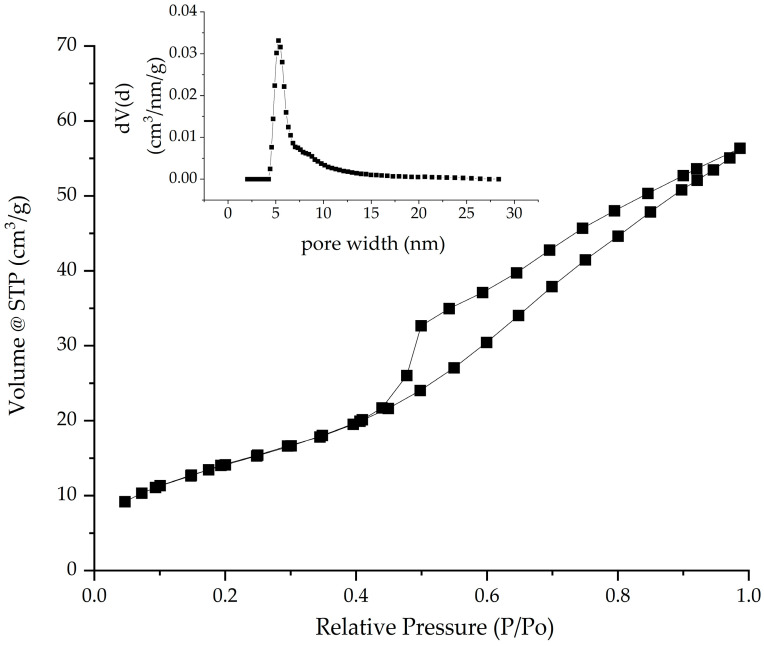
N_2_ adsorption–desorption isotherms and pore size distribution (inset) for SFZ material.

The morphology of the material is a necessary characteristic that indicates the influence of its adsorption capacity. Figure 4 shows the N_2_ adsorption–desorption isotherm and the pore size distribution specific to the obtained composite. By comparative analysis with IUPAC [57], it is observed that the SFZ sample shows the type IVa shape for the nitrogen adsorption–desorption isotherm.

The presence of H2b type hysteresis shows that the material consists mainly of mesopores. This material also shows inkbottle type pores, but the pore neck size is much larger. Following the pore distribution represented by the inset in Figure 4, using the DFT method (Density Functional Theory Calc. Model: N_2_ at 77 K on silica (cylinder pore, NLDFT equilibrium model)), it is observed that the pore size has an average value of ~5 nm, confirming that the material is mesoporous (i.e., with values located between 2 and 50 nm). After confirming that the material is mesoporous via the DFT method, the specific calculation for mesoporous materials was also performed by the BJH method (Barrett–Joyner–Halenda Model), indicating a similar value for the average pore diameter (~5 nm).

The total pore volume calculated from the last point of the isotherm on the adsorption branch at a pressure P/Po = 0.99 indicates a value of 0.087 cm^3^/g. The specific surface area of the material calculated by the BET method, evaluated in the range of 0.05–0.25 P/Po, indicates a value of 53 m^2^/g, with C constant at a value of 48.

The fractal dimension obtained by the Frenkel–Halsey–Hill model (FHH method) from the adsorption branch for the SFZ sample indicates a value of D = 1.8558. When the value of the materials is close to 2, the materials have a 2D surface, meaning that this material is smooth on the surface.

#### 2.1.5. Small-Angle X-Ray Scattering (SAXS) Measurements

**Figure 5:**
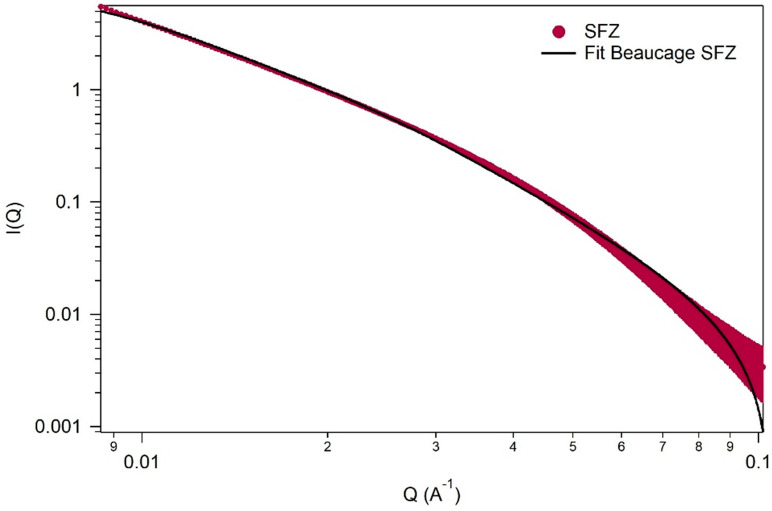
SAXS measurement for sample SFZ at room temperature in vacuum.

Analysis in the SAXS region (Figure 5) can provide us with essential information regarding the structure of materials, especially for nanocomposites with dimensions from 1 to 100 nm. To evaluate the results obtained from the SAXS region, one-level fits of the material were performed using the Beaucage function (i = 1) [58,59].

The parameters evaluated from the SAXS curves after adjustment show an Rg (gyration radius) value of 17.8 ± 0.4 nm. From the Rg value, the diameter of the particle size can be deduced if we assume that it is spherical, using the equation: D = 2.6 Rg. The particle diameter indicates a value of ~46 nm.

Compared with the literature [60,61], for pure fractal structure, the power exponent should be less than 4. In our case, the power low exponent, p, from the small q range indicates a value of 2.93 ± 0.02, demonstrating that this material is characterized by mass fractals. Comparing the data, it is observed that, in the case of Sku values obtained by AFM and D values obtained by the FHH method, both indicate that the material presents a mass fractality.

#### 2.1.6. Determination of Point of Zero Charge (pH_pZc_) for Adsorbent Particle

It is known that the pZc is the pH value where the net electrical charge on a surface is zero. The pZc is a crucial concept that influences the behavior of materials in the adsorption process [62].

**Figure 6:**
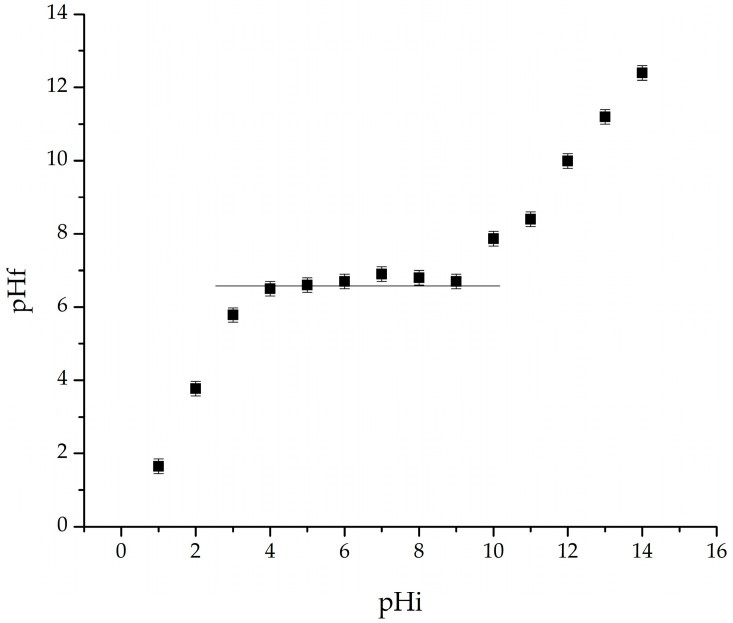
Point of Zero Charge (pH_pZc_) for SFZ material.

The experimental plot of pH_f_ (final pH) vs. pH_i_ (initial pH) is shown in Figure 6.

From Figure 6, it can be noted that the pH_pZc_ of the material is 6.7, which means that, at this pH, the surface charge of the adsorbent is zero. Once the pH of the solution is lower than the pH_pZc_, the surface charge of the adsorbent becomes positive due to the adsorption of excess H^+^ ions. On the other hand, the surface charge becomes negative due to the desorption of H^+^ ions [63]. Knowing the zero charge point of the adsorbent can provide information about attraction or repulsion phenomena between the adsorbent and the adsorbate, to establish the adsorption mechanism. The adsorption of La(III) on SFZ material is a complex process, involving physical interactions between the La(III) ion in solution and the surface of the SFZ material. The presumed mechanism consists of electrostatic interactions by which the La(III) ions, which are positively charged, are attracted to the solid surface, which may have a negative charge. This electrostatic attraction is strong for La(III) ions, with a charge of 3+.

### 2.2. Adsorption Studies

Adsorption is the operation of separating a component from a solution by retaining it on the surface of a solid (called the adsorbent). The optimization of La(III) recovery parameters by adsorption was studied, depending on the pH of the La(III) solution, the adsorbent dose, the contact time, the temperature, and the initial concentration of the La(III) solution. The experimental data obtained were processed and interpreted using mathematical models specific to the Langmuir, Freundlich, and Sips adsorption isotherms. Evaluation of the kinetic and thermodynamic parameters characteristic of the adsorption processes studied was also carried out. At the same time, the activation energy value was established.

#### 2.2.1. Adsorption Parameter Optimization for La(III) Recovery

**Figure 7:**
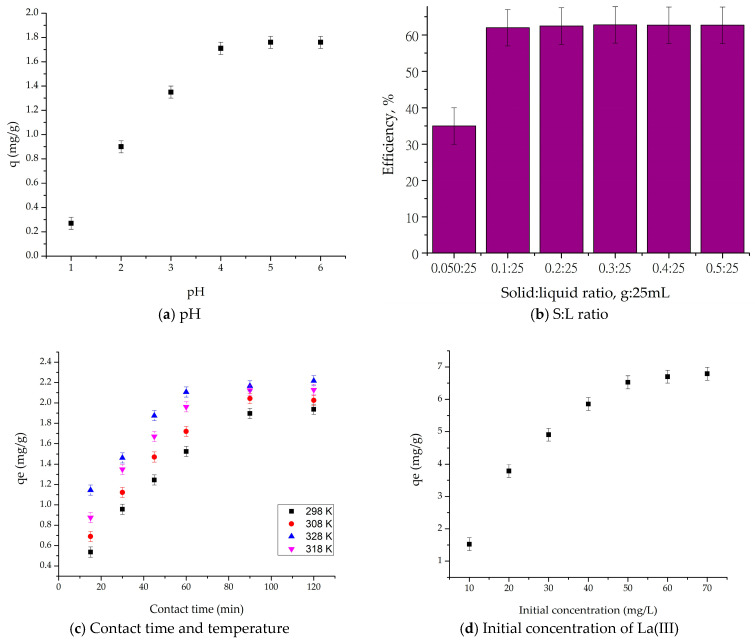
Adsorption parameter optimization for La(III) recovery.

The influence of the pH of the absorption medium was investigated for the absorption of La(III) ion on the SFZ material and is shown in Figure 7.

From Figure 7a, we can observe a favorable adsorption of the La(III) ion for pH values between 4 and 6; after this value, adsorption no longer exists because of the precipitation of La(III) at pH > 6. This is due to the fact that, at higher pH values, there is a competition for adsorption centers between hydroxyl ions formed on the surface of the material and La(III) species.

In order to establish the optimal S/L ratio for La(III) recovery, from the experimental data presented in Figure 7b it can be concluded that, up to a ratio of 0.1 g:25 mL, the efficiency of the adsorption process increases and, at higher ratios, the efficiency of the process remains approximately constant (62%).

Further studies aimed to establish the contact time and the optimal temperature (Figure 7c). Rapid adsorption can be observed in the first 90 min, after which the recovered amount reaches a maximum that does not change significantly until 120 min. Thus, up to 90 min, the adsorption capacity increases, after which it remains constant (qe = 1.52 mg/g for 298 K) regardless of the increase in time.

Another specific adsorption parameter is temperature. As temperature increases, the adsorption capacity increases, but not significantly enough to recommend that the recovery process proceed at a temperature higher than 298 K.

We can state that, following these studies, the optimal adsorption conditions are pH = 4–6; S:L ratio = 0.1 g:25 mL; contact time 90 min and temperature 298 K. Under these conditions, the equilibrium concentration and the maximum adsorption capacity were established. Thus, from Figure 7d, it can be concluded that, for an initial concentration of 60 mg La(III)/L, an adsorption capacity of 6.7 mg/g is obtained, considered as the maximum adsorption capacity in the present experimental conditions.

#### 2.2.2. Kinetic Studies

The Langmuir model describes adsorption in systems as follows: (i) a process that occurs on a homogeneous surface, where all adsorption sites are equivalent; (ii) a process by which a monolayer of adsorbed molecules is formed; (iii) a process in which there are no interactions between adsorbed molecules; (iv) adsorption reaches a dynamic equilibrium. The Freundlich model (i) describes adsorption on heterogeneous surfaces, where adsorption sites have different energies; (ii) adsorption can form several layers; (iii) is an empirical model, based on experimental data, not on a specific theory. The Sips model is a hybrid model that combines the characteristics of the Langmuir and Freundlich isotherms and that (i) describes adsorption on heterogeneous surfaces; (ii) is useful for modeling adsorption in complex systems; and (iii) is used to determine the maximum adsorption capacity [64]. The Ho and McKay (pseudo-second-order) model is a kinetic model that describes liquid-phase adsorption, is based on the pseudo-second-order kinetic equation and can be used to determine the adsorption rate constant [65]. The Lagergren (pseudo-first-order) model is another kinetic model that describes liquid-phase adsorption, is based on the pseudo-first-order kinetic equation and is simpler than the Ho and McKay model, but may be less accurate [65]. The Weber and Morris model describes intraparticle diffusion, an important process in adsorption on porous materials, and is used to determine the intraparticle diffusion coefficient and helps to understand the process of diffusion of the adsorbate into the pores of the adsorbent. These mathematical models are essential for understanding and optimizing adsorption processes. They allow the determination of important parameters, such as adsorption capacity, rate constant and diffusion coefficient. This information is crucial for the efficient design and operation of adsorption systems.

In this context, the kinetics of the adsorption process of La(III) on SFZ material was studied, using two kinetic model equations that could describe the process: the Lagergren model (pseudo-first-order) and the model proposed by Ho and McKay (pseudo-second-order). In order to distinguish whether film diffusion or intraparticle diffusion (IPD) is the rate-determining step, the experimental data obtained from the kinetic studies are fitted using the Weber and Morris model.

The linear forms of the three models are used to model the experimental data. The rate constant for the pseudo-first-order model is determined from the linear representation of *ln*(*qe* − *qt*) as a function of time, and the rate constant for the pseudo-second-order model is estimated from the linear representation of *t/qt* as a function of time.

For intraparticle diffusion to be the only rate-determining step, it is necessary that the plot of qt as a function of t_0.5_ be a curve shows the best possible linearity, passing through the origin (C = 0). Otherwise, both intraparticle diffusion and film diffusion influence the adsorption kinetics. A negative value of C also indicates that film diffusion influences the adsorption kinetics.

**Figure 8:**
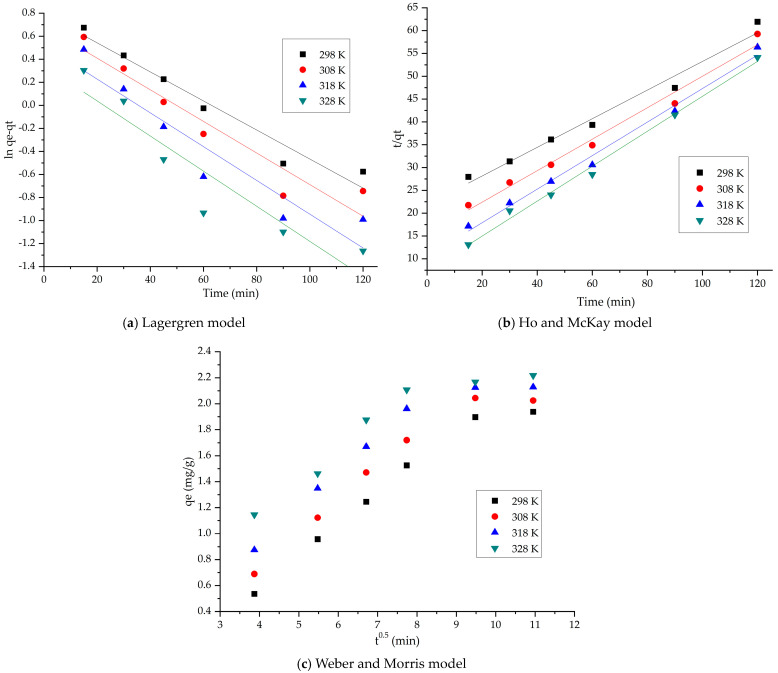
Kinetic studies for La(III) recovery by adsorption.

Depending on the values of the constants and the regression coefficients (R^2^) obtained, the kinetic model that best describes the adsorption process can be established. The data are presented in Figure 8 and Table 2.

Starting from the value of the regression coefficient R^2^, it can be stated that the adsorption process of La(III) on the SFZ material behaves, from a kinetic point of view, in a similar manner to that in the pseudo-second-order kinetic model (R^2^ > 0.99). In addition, qe,calc based on the pseudo-second-order isotherm model has values close to q_e,exp_.

At the same time, it is observed that the adsorption mechanism of La(III) is carried out in several stages, because the straight lines obtained by graphically representing the dependence of qt = f(t^1/2^) at different temperatures do not pass through the origin (C = 0). Thus, we can state that both intraparticle diffusion and film diffusion influence the adsorption kinetics. From the data presented in Table 2, it is observed that, with increasing temperature, the K_diff_ value also increases. It is also observed that, specific to stage 1, the diffusion constants are higher than the diffusion constants specific to stage 2, which allows us to state that stage 1 is the speed determinant and that, in stage 2, the process is slower [66].

#### 2.2.3. Thermodynamic Studies

**Figure 9:**
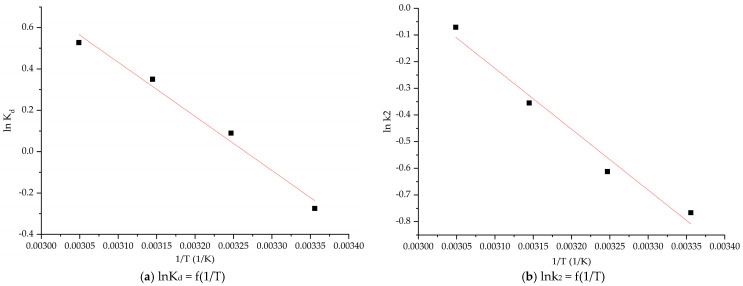
Thermodynamic study and Arrhenius plot for La(III) recovery by adsorption.

Thermodynamic parameters, such as the Gibbs free energy change (∆*G°*), the enthalpy change (∆*H°*) and the entropy change (∆*S°*), were studied to evaluate the feasibility and nature of the adsorption process. The enthalpy and entropy change values were calculated from the slope of the linear dependence lnKd = f(1/T) and these values are shown in Figure 9a and Table 3. From Table 3, the values of the Gibbs free energy changes are negative for the adsorbent material at all temperatures, indicating the feasibility and spontaneous nature of the adsorption process.

Based on the negative values of ∆*H°*, it can be said that the adsorption process of the La(III) ion on the adsorbent material is favorable and endothermic. The positive value of the standard entropy indicates a decrease in free spaces at the solid–liquid interface during the adsorption of La(III) on the material and also suggests that the system presents a disordered adsorption. From the graphical representation, lnk_2_ = f(1/T) is determined—Ea/R (Figure 9b).

Based on the experimental data, the activation energy was calculated at 2.45 kJ/mol, the regression coefficient being R^2^ = 0.9996. Since the activation energy is <40 kJ mol^−1^, it means that the adsorption process is of a physical nature [67].

#### 2.2.4. Equilibrium Studies

**Figure 10:**
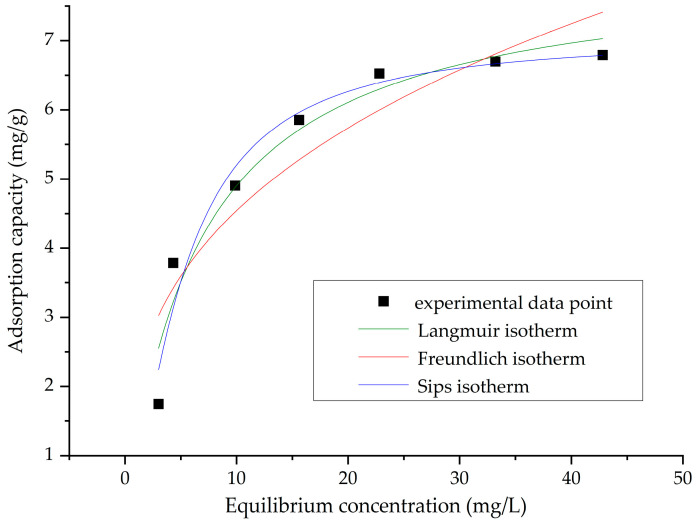
Equilibrium studies for La(III) recovery by adsorption.

The experimental data were processed using Langmuir, Freundlich, and Sips isotherms and are presented in Figure 10 and Table 4.

The Langmuir isotherm effectively describes the adsorption data, with regression coefficient values of 0.9374 in the established conditions (pH 4–6, S:L ratio = 0.1 g:25 mL, 60 min contact time, 298 K). It can be said that temperature is a favorable factor for the adsorption of the La(III) ion on SFZ material.

Another isotherm used to process the experimental data obtained from the adsorption of La(III) ion on SFZ material is the Freundlich isotherm. The regression coefficient, 0.8325, indicates that the model describes the adsorption mechanism least precisely.

The Sips isotherm was also used to describe the adsorption of La(III) ion on SFZ material. In this case, a good fit of the experimental data to the Sips isotherm model is observed, the value of the regression coefficient being 0.9451.

In order to better highlight the adsorption properties of the studied material, a comparative study of its adsorption capacity with other adsorbent materials available in the literature was carried out. The information found is presented in Table 5.

### 2.3. Regeneration Degree of Adsorbent

**Figure 11:**
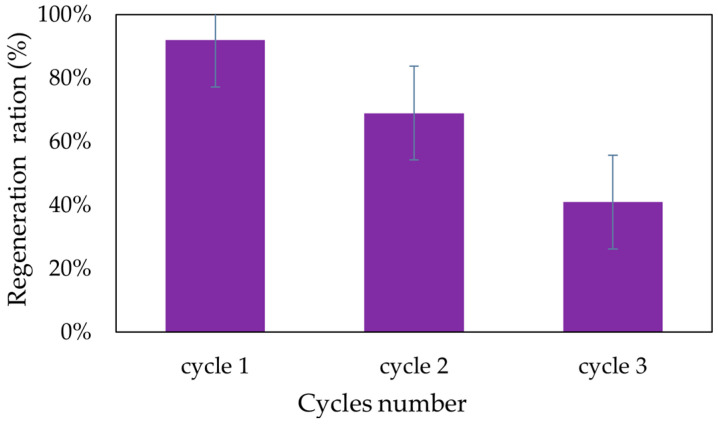
Regeneration cycles for SFZ material.

The regeneration capacity of adsorbent material is important for its economic viability and efficiency in practical applications. The study used a 5% HCl solution to regenerate the adsorbent material. Acidic solutions are often used to desorb metal ions due to their ability to disrupt the interactions between the adsorbent and the adsorbed species. The adsorbent material achieved a regeneration ratio of 92% (Figure 11).

It can be seen from the image that the SFZ material can be used for three adsorption/desorption cycles with good results. This indicates that a significant portion of the adsorption capacity is retained after exposure to the regeneration solution, making the material potentially suitable for repeated use.

## 3. Conclusions

In this work it was proposed to synthesis a xerogel based on SiO_2_, Fe_2_O_3_ doped with ZnO for the efficient recovery of La(III) from aqueous medium. The chosen synthesis method was the sol–gel method. The adsorbed material was physico-chemically characterized by various specific methods, such as TG–DTG, FT–IR, Raman, SEM, EDX, AFM and SAXS, and the evaluation of the mechanism of La(III) recovery by adsorption was established.

The SEM micrograph indicates the existence of particles with irregular geometric shapes and sizes ranging from 16 µm to 45 µm. From SAXS curve interpretation, the values obtained for the particle diameter indicate a value of ~46 nm. Most of the particles present are distributed separately throughout the area. The EDX spectrum confirms the presence of specific peaks of Si, Fe, Zn and O, certifying the formation of oxides SiO_2_, Fe_2_O_3_ and ZnO.

The specific surface area of the synthesized material was determined by the BET method that showed the mesopore structure of the material. Following the pore distribution, by the DFT method, it was observed that the pore size has an average value of ~5 nm, confirming that the material is mesoporous. The fractal dimension obtained by the FHH method (Frenkel–Halsey–Hill model) of the SFZ sample indicates that the material does not present roughness, the material being smooth on the surface.

To establish the optimal conditions for the recovery of La(III) from aqueous media by adsorption, studies were carried out, finding that, at pH 4–6, S:L ratio = 0.1 g:25 mL; contact time 90 min, and temperature 298 K for an initial concentration of 60 mg La(III)/L, the maximum adsorption capacity of the SFZ material can be obtained, being 6.7 mg/g.

The regeneration test highlights the fact that the material can be successfully regenerated at a 92% proportion and can be reused at least three times with good efficiency.

The adsorption of La(III) on SFZ material is a complex process, involving physical interactions between the La(III) ion in solution and the surface of the SFZ material. The presumed mechanism consists of electrostatic interactions by which the La(III) ions, which are positively charged, are attracted to the solid surface, which may have a negative charge. This electrostatic attraction is strong for La(III) ions with a charge of 3+. From a kinetic point of view, the mechanism of the adsorption process was established. The experimental data were modelled with a significant correlation coefficient according to the Lagergren model (second-pseudo-order) and, according to the Weber and Morris model (intraparticle diffusion), the adsorption was carried out in several stages, and the rate determinant is stage 1, the process being slower in stage 2. From a thermodynamic point of view, the adsorption process is spontaneous, endothermic, and occurs at the adsorbent–adsorbate interface. At the same time, the Sips model is that which best fits the experimental data.

In conclusion, the SFZ material obtained is an efficient adsorbent for the removal of La(III) from aqueous solutions.

## 4. Materials and Methods

### 4.1. Synthesis of SFZ

**Figure 12:**
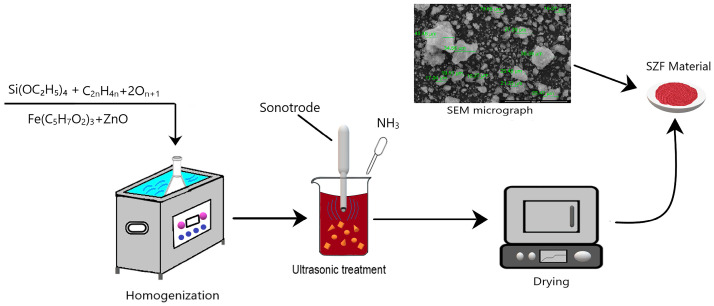
Synthesis method for SFZ.

A suggestive synthesis path for SFZ xerogel is presented in Figure 12.

The material synthesis method used in this study is sol–gel by sonication. The SiO_2_ precursor, obtained by dissolving 15 mL tetraethyl orthosilicate (TEOS) (Si(OC_2_H_5_)_4_, ≥99.0%, purchased from Sigma-Aldrich Chemie GmbH, Steinheim, Germany) in 20 mL absolute ethanol (Riedel-de Haen, 99.8% vol.), was added to the polyethylene glycol (PEG). The PEG solution was obtained by dissolving 5 mL polyethylene glycol, PEG 400 (Roth Rotipuran Ph.Eur, Karlsruhe, Germany) in 10 mL distilled water. For better homogenization, the reaction mass was homogenized for 10 min in an ultrasound bath (Sonorex Super 10 P Bandelin, Bandelin electronic GmbH and Co KG, Berlin, Germany), delivering an energy of 25,000 J with 15/15 pulse cycles, the frequency being 50 Hz at a maximum temperature of 328 K.

The Fe_2_O_3_ precursor obtained by dissolving 3.75 g of iron (III) acetylacetonate, Fe(acac)_3_ (Sigma-Aldrich, Chemie GmbH, Steinheim, Germany) in 10 mL absolute ethanol (Riedel-de Haen, 99.8% vol.) was added to the reaction mass, homogenizing for 10 min. Then, 0.1 g of ZnO (Merck Darmstadt, Germany) was added, continuing the homogenization.

The reaction mass obtained was moved to a sonotrode (VCX 750) where a final energy of ~25,000 J was introduced with an amplitude of 25%, a pulse of 15/15 and a maximum temperature of 55 °C. After 4 ultrasonication cycles, 1 mL of NH_3_ (Chimopar Trading SRL, Bucharest, Romania) was added, obtaining a solution, which before gelation had pH = 10. To obtain the xerogel, the obtained material was dried for 24 h in an oven (POL-EKO SLW53, Wodzisław, Poland) at 100 °C.

### 4.2. Characterization of SFZ

The thermal analysis was carried out using a TGA 5500 thermogravimetric analyzer (TA Instrument, New Castle, DE, USA). The sample with a mass of approximately 10 mg was placed in a Pt HT crucible of 150 µL. Measurement was carried out in a nitrogen atmosphere (20 mL/min), within a temperature range of 25–600 °C, with a heating rate of 10 °C/min. In order to obtain information about the structure of materials at the nano and meso scale, Small Angle X-ray Scattering (SAXS) (Xenocs Xeuss 3.0, Xenocs SAS, Grenoble, France) was used. The sample was placed in a vacuum at room temperature with a powder holder using Kapton and calibrated with silver behenate. The distance for measurement by the detector (Dectris) was 1.8m at very high flux using a Cu source. The fitting was performed using SAS profit software (SasView version 5.0.6.). After substruction of Kapton from the initial data, one-level fits of the material were performed using the Beaucage function (*i* = 1) with the following equation:

(1) $$I\left(q\right)=Bkgd+\sum_{i=1}^{N}G_{i}\exp\;\left(-\frac{q^{2}R_{g.i}^{2}}{3}\right)+\frac{{B_{i}[erf\;(qR_{g.i}/\sqrt{6})]}^{3P_{i}}}{q^{P_{i}}}$$

where

$I\left(q\right)\;$—scattered intensity;
$G_{i}$—gyration radius;
q—scattering variable;
$R_{g.i}\;$—radius of gyration of the mass fractal object;
$P_{i}\;$—Porod constant;
$B_{i}$—2*G_i_*/*R_g*.*i_*.

The nanoparticles produced were evaluated using physicochemical techniques, including Raman at room temperature and 514 nm laser excitation with Shamrock 500i Spectrograph, (Angor, UK).

In the next step, the produced material was analyzed by recording the AFM image using the Scanning Probe Microscopy Platform (MultiView-200 system, Nanonics Imaging Ltd., Jerusalem, Israel), in the intermittent mode in normal conditions (25 °C). This analysis was carried out by using a chromium-doped tip with a 20 nm radius and 30–40 kHz resonance. Atomic force microscopy (MultiView-200 system, Nanonics Imaging Ltd., Jerusalem, Israel) was used to quantify the material surface roughness.

Further, the xerogel was characterized by recording the Fourier transform infrared spectra (FT–IR) using a Nicole iS50 FT–IR Spectrometer Nicolet™ iS50 FT–IR Spectrometer (Donau, Austria).

The prepared adsorbent material was characterized by scanning electron microscopy (SEM), Thermo Fisher Scientific, Hillsboro, OR, USA, coupled with dispersive X-ray spectroscopy (EDX) (Thermo Fisher Scientific, Hillsboro, OR, USA), using a scanning electron microscope, FEI Quanta FEG 250 (Thermo Fisher Scientific, Hillsboro, OR, USA). The sample being in powder form, it does not require any preparation. The sample was mounted directly on a stub for analysis, ensuring a secure fixation to avoid movement.

Nitrogen desorption isotherms were determined at 77 K using a Nova 1200e (Quantachrome Instruments, Boynton Beach, FL, USA) device, and pore size distributions were derived with the Barrett–Joyner–Halenda (BJH) method; specific surface areas were determined using the Brunauer–Emmett–Teller (BET) method. Prior to the analyses, the probes were degassed in vacuum for 5 h at room temperature. The surface area for various synthesized batches were replicated in the same conditions and the results indicate values within the standard error of 5%.

The zero load point, pH_pZc_, was also determined by the method of bringing the studied system to equilibrium. An amount of 0.1 g of material was used for this study, which was mixed with 25 mL of 0.1 N KCl solution (Carl Roth GmbH + Co, Karlsruhe, Germany) at 200 rpm and a temperature of 298 K, using a water bath with thermostating and stirring (Julabo SW23 Seelbach, Germany). The pH of the KCl solutions was adjusted in the range of 1–14, using NaOH solution (Carl Roth GmbH + Co., Karlsruhe, Germany) with a concentration between 0.05 N and 2 N, or HNO_3_ solution (Carl Roth GmbH + Co, Karlsruhe, Germany) with a concentration between 0.05 N and 2 N. The samples were filtered and subsequently the pH of the resulting solution was determined using a pH meter (Mettler Toledo, SevenCompact, S 210, Schwerzenbach, Switzerland).

### 4.3. Adsorption Studies

#### 4.3.1. Solid/Liquid (S/L) Ratio Effect

To establish which is the optimum S/L ratio, the best adsorption efficiency had to be obtained. In this case, the quantity of adsorbent material (0.05, 0.1, 0.2, 0.3, 0.4, and 0.5 g of SFZ) was exchanged for a constant volume (25 mL) of La(III) (LaCl_3_·7H_2_O, Merck, Darmstadt, Germany), at 10 mg/L concentration.

Adsorptions were carried out using a shaker (Julabo SW23, Seelbach, Germany), at a temperature of 298 K, for a contact time of 60 min. Adsorption efficiency was determined using the following relation:

(2) $$Efficiency\;[\%]=\frac{Ci-Crez}{Ci}100$$

where

Ci—La(III) initial concentration (mg/L)
Crez—La(III) residual concentration (mg/L)

#### 4.3.2. pH Effect

pH influence over adsorption is determined by the formation of La(III) in the solution and by the nature of the SFZ surface. In the present paper, the influence of the solution pH was studied in the pH range 1 to 6. This did not work at pH > 6 because, at pH higher than 6, the La(III) begins to precipitate [75]. A total of 0.1 g of adsorbent material was mixed with 25 mL of La(III) solution with an initial concentration of 10 mg/L. Contact time was 60 min at temperature 298 K. pH of the solutions was adjusted using HCl or NaOH solutions with concentrations between 0.1 and 1 N.

Material adsorption capacity was evaluated using the following relation:

(3) $$q\;[\mathrm{mg}/g]=\frac{\left(Ci-Crez\right)V}{m}\;$$

where

q—adsorption capacity (mg/g)
Ci—La(III) initial concentration (mg/L)
Crez—La(III) residual concentration (mg/L)
V—solution volume (L)
m—SFZ material mass (g)

#### 4.3.3. Contact Time and Temperature Effect

In order to establish the influence of contact time and temperature over adsorption capacity, samples of 0.1 g were weighed, which were mixed with 25 mL solutions of La(III), having an initial concentration of 10 mg/L. Obtained samples were mixed for different time periods (15, 30, 45, 60, 90 and 120 min) at different temperatures (298, 308, 318 and 328 K), at 200 rpm.

#### 4.3.4. Initial Concentration Effect

The effect of initial concentration of La(III) ions over maximum adsorption capacity was examined in prepared solutions with initial concentrations of 10, 20, 40, 50, 60, and 70 mg/L. All adsorptions were carried out at pH 6, at 298 K, for a mixing time of 90 min.

La(III) was analyzed by atomic absorption spectroscopy, using a Varian Spectra AA 280 FS (Agilent, Santa Clara, CA, USA) atomic absorption spectrophotometer. The analysis was performed by emission with a mixture of acetylene and nitrous oxide, at a wavelength of 441.7 nm.

### 4.4. Modeling of Sorption Isotherms, Kinetics and Thermodynamics

#### 4.4.1. Adsorption Isotherms

Three models of equilibrium isotherms were used: (i) Langmuir isotherm which is based on the monolayer adsorption of the solute; (ii) Freundlich isotherm, which was originally developed for heterogeneous surfaces and (iii) Sips model, which is a model that combines the aforementioned models, Langmuir and Freundlich. 

The equilibrium isotherms were obtained by the graphical representation of *qe* = *f*(*Ce*), and the related parameters were obtained for each isotherm. The Langmuir and Freundlich constants were calculated using the linearized form of the resulting patterns after fitting.

Langmuir isotherm is [76]

(4) $$q_{e}=\frac{q_{L}K_{L}C_{e}}{1+K_{L}C_{e}}$$

where

$q_{e}$—equilibrium adsorption capacity (mg/g)
$C_{e}$—equilibrium concentration (mg/L)
$q_{L}$—Langmuir maximum adsorption capacity (mg/g)
$K_{L}$—Langmuir constant.

Freundlich isotherm is [77]:

(5) qe=KFCe1nF

where

$q_{e}$—maximum adsorption capacity (mg/g)
$C_{e}$—equilibrium concentration (mg/L)
$K_{F}$ and $n_{F}$—characteristic constants, which can be associated with the relative adsorption capacity of the adsorbent and the adsorption intensity.

Sips isotherm is [78]

(6) qe=qSKSCe1nS1+KSCe1nS

where

$q_{S}$—Sips maximum adsorption capacity (mg/g)
$K_{S}$—constant related to adsorbent adsorption capacity
$n_{S}$—heterogeneity factor.

#### 4.4.2. Adsorption Kinetics

The pseudo-first-order kinetic models, the Lagergren model, and pseudo-second-order kinetic models, or the Ho and McKay models, were applied to describe the kinetics of the adsorption of La(III) during the process.

Lagergren model is [79]

(7) $$\ln(q_{e}-q_{t})=lnq_{e}-k_{1}t$$

where

$q_{e}$—equilibrium adsorption capacity (mg/g)
$q_{t}$—adsorption capacity at a specific time *t* (mg/g)
$k_{1}—$ pseudo-first-order speed constant (min^−1^)
t—contact time (min)

Ho and McKay model is [80]

(8) $$\frac{t}{q_{t}}=\frac{1}{k_{2q_{e}^{2}}}+\frac{t}{q_{e}}$$

where

$q_{e}$—equilibrium absorption capacity (mg/g)
$q_{t}$—adsorption capacity at a specific time *t* (mg/g)
$k_{2}$—pseudo-second-order speed constant (g/mg·min)
t—contact time (min)

A linear plot of *ln*(*qe* − *qt*) = *f*(*t*) gives the equilibrium adsorption capacity, *qe_,calc_* as intercept and the slope gives the *k*_1_ rate constant for the pseudo-first-order kinetic model; a linear plot of *t*/*qt* = *f*(*t*) gives the slope as equilibrium adsorption capacity *qe,_calc_* and the intercept gives the *k*_2_ rate constant for the pseudo-second-order kinetic model.

Intraparticle diffusion is the movement of adsorbate species within the pores of the adsorbent material after their initial attachment to the surface. It is one of the key steps in the adsorption process, along with film diffusion (the transport of adsorbate from the bulk solution to the surface of the adsorbent) and surface adsorption (the interaction of the adsorbate with the adsorbent surface). The intra-particle diffusion was described by the Weber and Morris model [81]:

(9) $$q_{t}=k_{diff}\times {\;t}^{1/2}+C$$

where

$q_{t}$—adsorption capacity at t time (mg/g)
$k_{diff}$—speed constant for intraparticle diffusion (mg/g·min^1/2^)
C—constant correlated with the thickness of the liquid film surrounding the adsorbent particles.

The activation energy, *E_a_*, can give information about the nature of the adsorption process. whether it is physical or chemical. Thus, the activation energy *E_a_* was calculated using the Arrhenius equation and the velocity constant from the pseudo-order kinetic model two *k*_2_.

(10) $$k_{2}=Ae^{\frac{-E_{a}}{RT}}$$

where

$k_{2}$—rate constant (g/min·mg)
A—Arrhenius constant (g·min/mg)
$E_{a}$—activation energy (kJ/mol)
T—absolute temperature (K)
R—ideal gas constant (8.314 J/mol·K)

The activation energy of adsorption La (III) on SFZ was calculated from the equation of the line of graphical representation *ln k*_2_ = *f* (*1*/*T*).

#### 4.4.3. Thermodynamics of the Adsorption Process

Thermodynamics studies offer general information about the influence of temperature on the adsorption, and are suitable to predict the feasibility of the adsorption process. To determine how the La(III) ions adsorption proceeds on the surface of the adsorbent, Gibbs’ free energy (ΔG°) was calculated using the Gibbs–Helmholtz equation [82]:

(11) $$\Delta G^{0}=\Delta H^{0}-T\times \Delta S^{0}$$

where

$\Delta G^{0}$—free Gibbs energy standard variation (J/mol)
$\Delta H^{0}$—enthalpy standard variation (J/mol)
$\Delta S^{0}$—entropy standard variation (J/mol·K)
T—absolute temperature (K)

Standard variations of enthalpy and entropy were evaluated from linear dependence of *lnKd* = *f*(*1*/*T*) (linear form of Van’t Hoff equation), where *Kd* is the equilibrium constant, being calculated as the ratio between equilibrium adsorption capacity ($q_{e}$) and equilibrium concentration ($C_{e}$).

(12) $$lnKd=\frac{\Delta S^{o}}{R}-\frac{\Delta H^{o}}{RT}$$

where

Kd—constant of equilibrium
$\Delta S^{o}\;$—standard variation of entropy (J/mol·K)
$\Delta H^{o}\;$—standard variation of enthalpy (kJ/mol)
T—absolute temperature (K)
R —ideal gas constant (8.314 J/mol·K)

The equilibrium constant is given by the ratio of the *q_e_* and *C_e_*:

(13) $$Kd=\frac{q_{e}}{C_{e}}$$

### 4.5. Regeneration Experiments

#### 4.5.1. Regeneration Ratio

The SFZ material was regenerated with 5% HCl solution. A total of 5 g of SFZ exhausted material, following the adsorption process, were placed in contact with 100 mL of 5% HCl solution. Knowing the equilibrium concentration for La(III) from the studies carried out, i.e., C_ech_ = 60 mg/L, it is considered that this amount of La(III) is present on the material. The contact time was 2 h, and the temperature was 298 K. A magnetic stirrer (VEVOR SH-2 Agitator, Bacu, Romania) with a speed of 200 rpm was used. After 2 h, the material was filtered and La(III) analyzed in the HCl solution by atomic absorption spectroscopy (Agilent, Santa Clara, CA, USA), establishing C_rez_.

The regeneration ratio (RR) formula is

(14) $$RR\;\left[\%\right]=\frac{C_{ech}-C_{rez}}{C_{rez}}\times 100$$

where

$C_{ech}$—La(III) equilibrium concentration (mg/L)
$C_{rez}$—La(III) residual concentration (mg/L).

#### 4.5.2. Adsorption–Desorption Studies

To evaluate the long-term performance of the SFZ adsorbent material stability, repeated adsorption–desorption cycles were performed. A glass column with a diameter of 2 cm and a height of 6 cm was used, which was loaded with 5 g of material, over which a La(III) solution with a concentration of 60 mg/L was passed with a constant flow rate of 3 mL/min using a peristaltic pump (Heidolph Pump Drive 5206, Schwabach, Germany). Then, 25 mL sample sequences were taken, and the residual La(III) concentration was determined by atomic absorption spectrometry (Agilent, Santa Clara, CA, USA). At the same time, several desorption cycles were performed until the material was exhausted, thus establishing the maximum number of adsorption/desorption cycles. Desorption was performed with 5% HCl solution.

## Figures and Tables

**Table 1:** Values obtained from AFM analysis.

Sample Name	Ironed Area (µm^2^)	Sa (nm)	Sq (µm)	Sp (nm)	Sv (nm)	Sy (nm)	Sku	Ssk
SFZ	422.156	39.5616	53.9842	176.330	−147.405	323.735	4.0171	0.7808

**Table 2:** Kinetic parameters for the adsorption of La(III) onto SFZ.

Lagergren (Pseudo-First-Order) Model				
Temperature (K)	*q*_e,exp_ (mg/g)	*k*_1_ (min^−1^)	*q*_e,calc_ (mg/g)	R^2^
298	1.89	0.0102	2.05	0.9554
308	2.04	0.0131	1.96	0.9165
318	2.12	0.0147	1.68	0.8904
328	2.16	0.0152	1.40	0.8721
**Ho and McKay (Pseudo-Second-Order) Model**				
**Temperature (K)**	***q*_e,exp_** **(mg/g)**	***k*** **_2_** **(g/mg∙min)**	***q*** **_e,calc_** **(mg/g)**	**R^2^**
298	1.89	0.46	3.18	0.9784
308	2.04	0.54	2.90	0.9830
318	2.12	0.70	2.72	0.9901
328	2.16	0.93	2.61	0.9935
**Weber and Morris (IPD) Model**				
**Temperature (K)**		**K_diff_ (mg/g·min^1/2^)**	**C**	**R^2^**
298		0.156	0.151	0.9548
308		0.181	0.349	0.9168
318		0.198	0.452	0.8673
328		0.207	0.676	0.8365

**Table 3:** Thermodynamic parameters for adsorption of La(III) onto SFZ.

Δ*H*° (kJ/mol)	Δ*S*° (J/mol∙K)	Δ*G*° (kJ/mol)				R^2^
21.76	71.06	298 K	308 K	318 K	328 K	0.9839
		−21.1	−21.8	−22.5	−23.2	

**Table 4:** Parameters of isotherm model for adsorption of La(III) onto SFZ.

Langmuir Isotherm			
*q*_m,exp_ (mg/g)	*K*_L_ (L/mg)	*q*_L_ (mg/g)	*R* ^2^
6.7	0.153	8.1	0.9374
**Freundlich Isotherm**			
*K*_F_ (mg/g)	1/*n*_F_		*R* ^2^
2.09	0.33		0.8325
**Sips Isotherm**			
*K*_S_	*q*_S_ (mg/g)	1/*n*_S_	*R* ^2^
0.09	7.07	0.4	0.9451

**Table 5:** Comparative study of the adsorption capacity of the SFZ material with other adsorbent materials available in the literature.

Materials	Adsorption Capacities, mg/g	References
Halloysite	5.65	[68]
Aliquat-336 impregnated onto Amberlite XAD-4	3.29	[69]
Nanoporous aluminosilicates	1.25	[70]
Neem sawdust	2.3	[71]
Magnetite-pectin-chitosan	8.17	[72]
Hydroxyapatite	0.25	[73]
GA-g-PAM/SiO_2_	7.9	[74]
SFZ	6.7	This paper

## Data Availability

The original contributions presented in this study are included in the article. Further inquiries can be directed to the corresponding author.

## References

[1] Lee J. (2018). Pathways for greening the supply of rare earth elements in China. Nat. Sustain..

[2] Cockell C.S., Santomartino R., Finster K., Waajen A.C., Eades L.J., Moeller R., Rettberg P., Fuchs F.M., Van Houdt R., Leys N. (2020). Space station biomining experiment demonstrates rare earth element extraction in microgravity and Mars gravity. Nat. Commun..

[3] Weng Z., Jowitt S., Mudd G., Haque N. (2015). A detailed assessment of global rare earth element resources: Opportunities and challenges. Econ. Geol..

[4] Balaram V. (2019). Rare earth elements: A review of applications, occurrence, exploration, analysis, recycling, and environmental impact. Geosci. Front..

[5] Unal Yesiller S., Eroğlu A.E., Shahwan T. (2013). Removal of aqueous rare earth elements (REEs) using nano-iron based materials. J. Ind. Eng. Chem..

[6] Oruji S., Khoshbin R., Karimzadeh R. (2019). Combination of precipitation and ultrasound irradiation methods for preparation of lanthanum-modified Y zeolite nano-catalysts used in catalytic cracking of bulky hydrocarbons. Mater. Chem. Phys..

[7] Dhanuskar S., Naik S.N., Pant K.K. (2025). Catalytic cracking and deoxygenation of cottonseed oil to yield light olefins over lanthanum-impregnated zeolite catalysts. Sustain. Energy Fuels.

[8] Jain P., Tibrewal A., Krishnakumar V., Barathraj R.K. (2020). Design, analysis and fabrication of lanthanum strontium manganite catalytic converter. IOP Conf. Ser. Mater. Sci. Eng..

[9] Furfori S., Bensaid S., Russo N., Fino D. (2009). Towards practical application of lanthanum ferrite catalysts for NO reduction with H2. Chem. Eng. J..

[10] Sicius H., Sicius H. (2024). Rare earth metals: Lanthanides and third subgroup. Handbook of the Chemical Elements.

[11] Alshehri S.A., Almotawa R.M., Sadeq M.S., Alzahrani J.A., Mahmoud A.E.-R., Amin H.Y. (2024). Unlocking the secrets of colored glass: Structural-optical behaviors and non-linear optical properties of La3+ and Fe3+ ions-doped borates. Opt. Mater..

[12] Mrabet C., Boukhachem A., Amlouk M., Manoubi T. (2016). Improvement of the optoelectronic properties of tin oxide transparent conductive thin films through lanthanum doping. J. Alloys Compd..

[13] Sayyed M.I., Rammah Y.S., Laariedh F., Abouhaswa A.S., Badeche T.B. (2020). Lead borate glasses doped by lanthanum: Synthesis, physical, optical, and gamma photon shielding properties. J. Non-Cryst. Solids.

[14] Jeevarajan J.A., Lamb J., Sgobba T., Musgrave G.E., Johnson G., Kezirian M.T. (2023). Chapter 21-Battery safety. Safety Design for Space Systems.

[15] Na C.K., Park H.J. (2010). Defluoridation from aqueous solution by lanthanum hydroxide. J. Hazard. Mater..

[16] Wang Y., Guo C., Zhang L., Lu X., Liu Y., Li X., Wang Y., Wang S. (2022). Arsenic oxidation and removal from water via core–shell MnO_2_@La(OH)_3_ nanocomposite adsorption. Int. J. Environ. Res. Public Health.

[17] Biddau R., Bensimon M., Cidu R., Parriaux A. (2009). Rare earth elements in groundwater from different alpine aquifers. Geochemistry.

[18] Kulkarni P., Chellam S., Fraser M.P. (2006). Lanthanum and lanthanides in atmospheric fine particles and their apportionment to refinery and petrochemical operations in Houston, TX. Atmos. Environ..

[19] Guan W., Zhang J., Liu Q. (2024). Quantitative evaluation of anthropogenic sources and health risks of rare earth elements in airborne particulate matter. Sci. Total Environ..

[20] Arienzo M., Ferrara L., Trifuoggi M., Toscanesi M. (2022). Advances in the fate of rare earth elements, REE, in transitional environments: Coasts and estuaries. Water.

[21] Neira P., Romero-Freire A., Basallote M.D., Qiu H., Cobelo-García A., Cánovas C.R. (2022). Review of the concentration, bioaccumulation, and effects of lanthanides in marine systems. Front. Mar. Sci..

[22] Herrmann H., Nolde J., Berger S., Heise S. (2016). Aquatic ecotoxicity of lanthanum—A review and an attempt to derive water and sediment quality criteria. Ecotoxicol. Environ. Saf..

[23] Fraile P., Cacharro L.M., Garcia-Cosmes P., Rosado C., Tabernero J.M. (2011). Encephalopathy caused by lanthanum carbonate. Nephrol. Dial. Transplant. Plus.

[24] Rim K.T., Koo K.H., Park J.S. (2013). Toxicological evaluations of rare earths and their health impacts to workers: A literature review. Saf. Health Work..

[25] Kampmann J., Hansen N.P., Ørsted Schultz A.N., Brandt A.H., Brandt F. (2022). Lanthanum carbonate opacities-a systematic review. Diagnostics.

[26] Betha R., Selvam V., Blake D.R., Balasubramanian R. (2011). Emission characteristics of ultrafine particles and volatile organic compounds in a commercial printing center. J. Air Waste Manag. Assoc..

[27] Dufresne A., Krier G., Muller J.F., Case B.W., Perrault G. (1994). Lanthanide particles in the lung of a printer. Sci. Total environment.

[28] Hernández-Campos M., Polo A.M.S., Sánchez-Polo M., Rivera-Utrilla J., Berber-Mendoza M.S., Andrade-Espinosa G., López-Ramón M.V. (2018). Lanthanum-doped silica xerogels for the removal of fluorides from waters. J. Environ. Manag..

[29] Shariatinia Z., Esmaeilzadeh A. (2019). Hybrid silica aerogel nanocomposite adsorbents designed for Cd(II) removal from aqueous solution. Water Environ. Res..

[30] Vareda J.P., Durães L. (2019). Efficient adsorption of multiple heavy metals with tailored silica aerogel-like materials. Environmental Technology.

[31] Zhang B., Xu L., Zhao Z., Peng S., Yu C., Zhang X., Zong Y., Wu D. (2022). Enhanced phosphate removal by nano-lanthanum hydroxide embedded silica aerogel composites: Superior performance and insights into specific adsorption mechanism. Sep. Purif. Technol..

[32] Akti F., Balci S. (2023). Silica xerogel and iron doped silica xerogel synthesis in presence of drying control chemical additives. Mater. Chem. Phys..

[33] Parashar M., Shukla V.K., Singh R. (2020). Metal oxides nanoparticles via sol–gel method: A review on synthesis, characterization and applications. J. Mater. Sci. Mater. Electron..

[34] Raileanu M., Crisan M., Petrache C., Crisan D., Zaharescu M. (2003). Fe_2_O_3_-SiO_2_ nanocomposites obtained by different sol-gel routes. J. Optoelectron. Adv. Mater..

[35] Jitianu A., Raileanu M., Crisan M., Predoi D., Jitianu M., Stanciu L., Zaharescu M. (2006). Fe3O4–SiO2 nanocomposites obtained via alkoxide and colloidal route. J. Sol-Gel Sci. Technol..

[36] Kumar R.V., Koltypin Y., Cohen Y., Cohen Y., Aurbach D., Palchik O., Felner I., Gedanken A. (2000). Preparation of amorphous magnetite nanoparticles embedded in polyvinyl alcohol using ultrasound radiation. J. Mater. Chem..

[37] Ianasi C., Costisor O., Putz A.-M., Lazau R., Negrea A., Niznansky D., Sacarescu L., Savii C. (2016). Low temperature superparamagnetic nanocomposites obtained by Fe(acac)_3_-SiO_2_-PVA hybrid xerogel thermolysis. Process. Appl. Ceram..

[38] Pal B., Sharon M. (2000). Preparation of iron oxide thin film by metal organic deposition from Fe(III)-acetylacetonate: A study of photocatalytic properties. Thin Solid Film..

[39] Ismail H.M. (1991). A thermoanalytic study of metal acetylacetonates. J. Anal. Appl. Pyrolysis.

[40] Putz A.-M., Ianăși C., Dudás Z., Coricovac D., Watz C., Len A., Almásy L., Sacarescu L., Dehelean C. (2020). SiO_2_-PVA-Fe(acac)_3_ hybrid based superparamagnetic nanocomposites for nanomedicine: Morpho-textural evaluation and in vitro cytotoxicity assay. Molecules.

[41] Panczer G., De Ligny D., Mendoza C., Gaft M., Seydoux-Guillaume A.-M., Wang X. (2012). Raman and fluorescence. Raman Spectrosc. Appl. Earth Sci. Cult. Herit..

[42] Cai J., Chen S., Ji M., Hu J., Ma Y., Qi L. (2014). Organic additive-free synthesis of mesocrystalline hematite nanoplates via two-dimensional oriented attachment. CrystEngComm.

[43] Mladin G., Ciopec M., Negrea A., Duteanu N., Negrea P., Ianasi P., Ianași C. (2022). Silica-iron oxide nanocomposite enhanced with porogen agent used for arsenic removal. Materials.

[44] Ianăşi C., Ianăşi P., Negrea A., Ciopec M., Ivankov O.I., Kuklin A.I., Almásy L., Putz A.-M. (2021). Effects of catalysts on structural and adsorptive properties of iron oxide-silica nanocomposites. Korean J. Chem. Eng..

[45] Matusoiu F., Negrea A., Nemes N.S., Ianasi C., Ciopec M., Negrea P., Duteanu N., Ianasi P., Duda-Seiman D., Muntean D. (2022). Antimicrobial perspectives of active SiO_2_FexOy/ZnO composites. Pharmaceutics.

[46] Lenza R.F., Vasconcelos W.L. (2001). Structural evolution of silica sols modified with formamide. Mater. Res..

[47] Motoc S., Ianasi C., Anamaria B., Delcioiu C., Sacarescu L., Lacrămă A.-M., Manea F. (2021). Humic acid removal from water by sorption and photocatalysis under Vis irradiation using Fe_2_O_3_/silica nanocomposite. Environ. Eng. Manag. J..

[48] Ivetic T., Dimitrievska M., Gúth I., Dacanin L., Lukic-Petrovic S. (2012). Structural and optical properties of europium-doped zinc oxide nanopowders prepared by mechanochemical and combustion reaction methods. J. Res. Phys..

[49] Gandomi F., Vakili M., Darugar V., Takjoo R., Tayyari S. (2021). Optimized molecular geometry, vibrational analysis, and Fe-O bond strength of Tris(α-cyanoacetylacetonate)iron(III):An experimental and theoretical study. J. Mol. Struct..

[50] Geisler T., Dohmen L., Lenting C., Fritzsche M.B.K. (2019). Real-time in situ observations of reaction and transport phenomena during silicate glass corrosion by fluid-cell Raman spectroscopy. Nat. Mater..

[51] Santillán J.M.J., Muñetón Arboleda D., Coral D.F., Fernández van Raap M.B., Muraca D., Schinca D.C., Scaffardi L.B. (2017). Optical and magnetic properties of Fe nanoparticles fabricated by femtosecond laser ablation in organic and inorganic solvents. ChemPhysChem.

[52] Uma K., Chen S.W., Arjun N., Pan G.T., Yang T.C. (2018). The production of an efficient visible light photocatalyst for CO oxidation through the surface plasmonic effect of Ag nanoparticles on SiO_2_@α-Fe_2_O_3_ nanocomposites. RSC Adv..

[53] Bajpai A.K., Bhatt R., Katare R. (2016). Atomic force microscopy enabled roughness analysis of nanostructured poly (diaminonaphthalene) doped poly (vinyl alcohol) conducting polymer thin films. Micron.

[54] Maksumov A., Vidu R., Palazoglu A., Stroeve P. (2004). Enhanced feature analysis using wavelets for scanning probe microscopy images of surfaces. J. Colloid Interface Sci..

[55] Bazaka K., Jacob M.V., Truong V.K., Crawford R.J., Ivanova E.P. (2011). The effect of polyterpenol thin film surfaces on bacterial viability and adhesion. Polymers.

[56] Sedlaček M., Gregorčič P., Podgornik B. (2017). Use of the roughness parameters Ssk and Sku to control friction—A method for designing surface texturing. Tribol. Trans..

[57] Thommes M., Kaneko K., Neimark A.V., Olivier J.P., Rodriguez-Reinoso F., Rouquerol J., Sing K.S.W. (2015). Physisorption of gases, with special reference to the evaluation of surface area and pore size distribution (IUPAC Technical Report). Pure Appl. Chem..

[58] Beaucage G. (1995). Approximations leading to a unified exponential/power-law approach to small-angle scattering. J. Appl. Crystallogr..

[59] Beaucage G. (1996). Small-angle scattering from polymeric mass fractals of arbitrary mass-fractal dimension. J. Appl. Crystallogr..

[60] Savii C., Almásy L., Ionescu C., Székely N.K., Enache C., Popovici M.I., Sora I., Nicoara D., Savii G., Resiga D. (2009). Mesoporous silica matrices derived from sol-gel process assisted by low power ultrasonic activation. Proc. Appl. Cer..

[61] Anitas E., López-Ruiz R. (2017). Small-angle scattering from mass and surface fractals. Complexity in Biological and Physical Systems—Bifurcations, Solitons and Fractals.

[62] Kosmulski M. (2020). The pH dependent surface charging and points of zero charge. Adv. Colloid Interface Sci..

[63] Al-Maliky E.A., Gzar H.A., Al-Azawy M.G. (2021). Determination of point of zero charge (PZC) of concrete particles adsorbents. IOP Conf. Ser. Mater. Sci. Eng..

[64] He Z., Ren B., Hursthouse A., Wang Z. (2020). Efficient removal of Cd(II) using SiO_2_-Mg(OH)_2_ nanocomposites derived from sepiolite. Int. J. Environ. Res. Public Health.

[65] Ungureanu E., Carmenica Doina J., Trofin A., Fortuna M., Ungureanu O., Ariton A., Trinca L.C., Brezuleanu S., Popa V. (2022). Use of Sarkanda Grass lignin as a possible adsorbent for As (III) from aqueous solutions—Kinetic and equilibrium studies. Cellul. Chem. Technol..

[66] Zhang S., Ning S., Liu H., Zhou J., Wang S., Zhang W., Wang X., Wei Y. (2020). Highly-efficient separation and recovery of ruthenium from electroplating wastewater by a mesoporous silica-polymer based adsorbent. Microporous Mesoporous Mater..

[67] Zhang Y., Yu F., Cheng W., Wang J., Ma J. (2017). Adsorption equilibrium and kinetics of the removal of ammoniacal nitrogen by zeolite X/activated carbon composite synthesized from elutrilithe. J. Chem..

[68] He Q., Chen X., Gong S., Huang L., Xiao Y. (2024). Adsorption behaviors and mechanisms of lanthanum ions and exchanger cations at halloysite-solution interface: Perspectives from electrical double layer model and spectral analyses. J. Mol. Liq..

[69] El-Sofany E.A. (2008). Removal of lanthanum and gadolinium from nitrate medium using Aliquat-336 impregnated onto Amberlite XAD-4. J. Hazard. Mater..

[70] Sepehrian H., Cheraghali R., Rezaei P., Abdi H. (2011). Adsorption behavior of lanthanum on modified mesoporous aluminosilicates. Int. J. Ind. Chem..

[71] Varshini C., Das N. (2014). Relevant approach to assess the performance of biowaste materials for the recovery of Lanthanum (III) from aqueous medium. Res. J. Pharm. Biol. Chem. Sci..

[72] Chaibou Yacouba A.-R., Oral A.E., Sert S., Kaptanoglu I.G., Natatou I., Yusan S., Aytas S. (2024). Removal of lanthanum and cerium from aqueous solution using chitosan-functionalized magnetite-pectin. Discov. Water.

[73] Granados-Correa F., Vilchis-Granados J., Jiménez-Reyes M., Quiroz-Granados L.A. (2013). Adsorption behaviour of La(III) and Eu(III) ions from aqueous solutions by hydroxyapatite: Kinetic, isotherm, and thermodynamic studies. J. Chem..

[74] Iftekhar S., Srivastava V., Casas A., Sillanpää M. (2018). Synthesis of novel GA-g-PAM/SiO_2_ nanocomposite for the recovery of rare earth elements (REE) ions from aqueous solution. J. Clean. Prod..

[75] Patnaik P. (2003). Handbook of Inorganic Chemicals.

[76] Langmuir I. (1918). The adsorption of gases on plane surfaces of glass, mica and platinum. J. Am. Chem. Soc..

[77] Freundlich H. (1907). Über die adsorption in lösungen. Z. Für Phys. Chemie.

[78] Sips R. (1948). On the structure of a catalyst surface. J. Chem. Phys..

[79] Lagergren S. (1898). About the theory of so-called adsorption of soluble substances. K. Sven. Vetenskapsakademiens Handl..

[80] Ho Y.-S. (2006). Review of second-order models for adsorption systems. J. Hazard. Mater..

[81] Weber W.J., Morris J.C. (1964). Equilibria and capacities for adsorption on carbon. J. Sanit. Eng. Div..

[82] Atkins P.W., De Paula J., Keeler J. (2023). Atkins’ Physical Chemistry.

